# Apolipoprotein L1 (APOL1) Variants (Vs) a possible link between Heroin-associated Nephropathy (HAN) and HIV-associated Nephropathy (HIVAN)

**DOI:** 10.3389/fmicb.2015.00571

**Published:** 2015-06-09

**Authors:** Xiqian Lan, T. K. S. Rao, Praveen N. Chander, Karl Skorecki, Pravin C. Singhal

**Affiliations:** ^1^Hofstra North Shore-LIJ School of MedicineLong Island, NY, USA; ^2^Department of Medicine, State University of New York Downstate Medical CenterBrooklyn, NY, USA; ^3^Department of Pathology, New York Medical CollegeValhalla, NY, USA; ^4^Technion Institute of Technology and Rambam Medical CenterHaifa, Israel

**Keywords:** APOL1, heroin nephropathy, HIV-associated nephropathy, podocytes, opiates

## Abstract

In 1970s, Heroin-associated Nephropathy (HAN), one form of focal and segmental glomerulosclerosis (FSGS), was a predominant cause of End-stage Kidney Disease (ESKD) in African-Americans (AAs). In 1980s, with the surge of Acquired Immune Deficiency Syndrome (AIDS) in AAs, HAN more or less disappeared, and the incidence of Human Immunodeficiency Virus associated Nephropathy (HIVAN) markedly increased. Recent studies in AAs have identified APOL1 variants (Vs) as a major risk factor for the development and progression of non-diabetic kidney diseases including idiopathic FSGS and hypertension-attributed nephrosclerosis. These observations have also offered partial insights into the mechanisms of development, and higher rate of occurrence of both HAN and HIVAN in AAs. AAs with APOL1Vs develop idiopathic FSGS at four-fold higher rate compared to European Americans (EAs). Similarly, HIV infected AAs with APOL1Vs (if not on antiviral therapy), risk a 50% (10-fold greater) chance of developing HIVAN. It has been suggested that APOL1Vs expression may render podocytes more vulnerable to various types of injury: bacterial, viral, and others. However, in addition to genetic variants, additional factors such as persistence of a second hit may determine the nature and severity of glomerular disease. In patients with HAN, heroin or contaminants may have been the offending second insult(s) which caused renal disease in susceptible AA patients. In the 80's, since heroin-induced second hit was neither consistent nor sustained (depending on drug availability in the street), the disease was masked or replaced HIV infected patients (especially in untreated subjects), by an overwhelming second hit by the virus which was both intense as well as persistent. It appears that APOL1Vs may be one of the links between the disappearance of HAN and emergence of HIVAN in AA patients.

## Introduction

There has been an explosive growth in illicit drug use in general and in the USA in particular during the last past 45 years. Although being only 4.6% of the world's population, Americans consume 80% of the global opiates supply and 99% of the global hydrocodone supply (Catalanom et al., 2011). According to CDC, 40% of HIV-infected individuals in the United States are associated with the use of injection drugs; similarly, among injection drug users, 40–45% are HIV infected (CDC, 2010). Thus, use of drugs in patients with HIV infection is an unresolved problem of high relevance. Since factors associated with heroin addiction such as bacterial and viral infections carry the potential to contribute to the development of renal lesions, it is not clear whether it is heroin or its metabolite, morphine, or associated adulterants which are responsible for the entity morphologically described as HAN (Kimmel and Moore, 1997; Freedman et al., 1999; Kimmel et al., 2001; Norris et al., 2001; Satko and Freedman, 2004).

The number of cases of “heroin nephropathy” has been decreasing over the years, despite the increased prevalence of heroin use. Although focal glomerulosclerosis has been a predominant pathological feature in patients with heroin abuse in several studies (Salomon et al., 1972; Friedman et al., 1974; Rao et al., 1977; Friedman and Rao, 1995), but other studies have not shown any uniform pathologic presentation (McGinn et al., 1970; Kilcoyne et al., 1972; Grisham et al., 1976; Llach et al., 1979; Cunningham et al., 1980, 1983; Dettmeyer et al., 1998; do Sameiro Fario et al., 2003). It is not clear whether heroin is contributing to renal lesions in these patients or whether nephropathy is related to demographic, socioeconomic, or genetic factors of individual users (Kimmel and Moore, 1997; Freedman et al., 1999; Kimmel et al., 2001; Norris et al., 2001; Satko and Freedman, 2004).

Focal segmental glomerulosclerosis (FSGS) is the most common type of renal lesions in AAs (Kitiyakara et al., 2003). Genetic factors do play a role in the development of FSGS but their role has been debated for a long time (Devarajan and Spitzer, 2002; Tucker, 2002; Kitiyakara et al., 2003, 2004; Kopp and Winkler, 2003; Winn, 2003; Andreoli, 2004; Orloff et al., 2005). On that account it has been suggested that that black heroin users with FSGS have a genetic susceptibility to the development of renal disease, unrelated to the drug use. Thus, it is not clear whether development of FSGS in drug users is primarily related to genetics or to the drug use. Alternatively, genetic may be a first hit and drug use might be the second hit. Moreover, besides FSGS several other types of renal lesions are associated with heroin use (McGinn et al., 1970; Kilcoyne et al., 1972; Grisham et al., 1976; Llach et al., 1979; Cunningham et al., 1980, 1983; Dettmeyer et al., 1998; do Sameiro Fario et al., 2003).

### Heroin-associated nephropathy (HAN)

In early 1970s, many clinicians observed excessive urinary protein excretion and renal functional abnormalities in heroin addicts. Rao et al. described the natural history of renal disease in heroin addicts who presented with massive proteinuria and focal segmental glomeulosclerosis (FSGS) which progressed to end stage kidney disease (ESKD) in months, and coined the term Heroin-Associated Nephropathy (HAN) (Kilcoyne et al., 1972; Rao et al., 1974, 1977). These patients were mostly young men predominantly of African American (AA) descent residing in large Metropolitan areas (Kilcoyne et al., 1972; Rao et al., 1974, 1977; Cunningham et al., 1983; Friedman, 1983; May et al., 1986). An increased risk for ESKD with an odds ratio of 19.1 was noted for heroin users vs. non-users (Cunningham et al., 1980). In addition to genetic predilection, occurrence of HAN was attributed to bacterial or viral toxins and street contaminants (Rao et al., 1977; Cunningham et al., 1980, 1983). HAN occurrence peaked in late 70's and early 80's and accounted for large number of young AA men with ESKD on maintenance dialysis (Cunningham et al., 1983). Renal involvement in heroin addiction manifested in multiple forms including FSGS, amyloidosis (in subcutaneous heroin addicts), endocarditis-associated glomerulonephritis, hepatitis-related glomerulonephritis, and acute renal failure from rhabdomyolysis (Kilcoyne et al., 1972; Rao et al., 1977; Cunningham et al., 1980, 1983; Friedman, 1983; Baldwin et al., 1993; Dettmeyer et al., 1998).

Other investigators found no specific histologic or immunofluorescence pattern in heroin users with renal disease (Llach et al., 1979). They described a spectrum of disease pattern including FSGS, minimal change disease, MPGN, mesangial proliferation, dysproteinemias, and diabetic nephropathy (Llach et al., 1979).

Unfortunately, there is no prospective controlled trial or well-designed epidemiologic studies have been carried out to confirm the role of heroin in the development of heroin nephropathy. Previous studies have been conducted predominantly on hospital-admitted patients and they may have several other confounding factors, which might have been missed.

### Opiates and kidney disease

Clinical studies indicated that opiate addicts are at an increased risk for progressive chronic kidney disease (Baldwin et al., 1993; Perneger et al., 2001). Since mesangial expansion is considered to be a precursor of focal glomerulosclerosis, we evaluated the effect of opiates on mesangial cell proliferation and matrix synthesis. *In vitro* studies have shown that morphine (a metabolite of heroin) directly stimulates proliferation of glomerular epithelial cells, mesangial cells, fibroblasts, and medullary interstitial cells (Singhal et al., 1992, 1997, 1998; Patel et al., 2003a). Morphine at higher concentration inhibited growth of mesangial cells, fibroblasts, and glomerular epithelial cells (Singhal et al., 1992, 1997, 1998; Patel et al., 2003a). It stimulates the production of superoxide by macrophages and nitric oxide by mesangial cells (Singhal et al., 1994; Kapasi et al., 2000). Morphine exerts a bimodal effect on heme oxygenase activity in glomerular epithelial cells, macrophages, and mesangial cells (Patel et al., 2003b) and also promoted macrophage apoptosis through the generation of transforming growth factor-β and reactive oxygen species (Singhal et al., 2000). Morphine-induced macrophage activity stimulated mesangial cell proliferation (Singhal et al., 1996a). *In vivo* studies, morphine inhibited matrix proteinases and promoted accumulation of matrix and IgG complexes in the mesangium (Sagar et al., 1994; Singhal et al., 1996a). Morphine also enhanced accumulation of IgG complexes in the mesangium of rats with anti-thymoglobulin-induced kidney injury (Pan and Singhal, 1994; Singhal et al., 1996b); additionally, it stimulated deposition of ferritin-antiferritin complexes in glomerular mesangium of rats (Singhal et al., 1995). Short-term administration of morphine invoked podocyte injury in the form of effacement of foot processes and down regulation of nephrin expression by podocytes (Lan et al., 2013). Morphine promoted mesangial cell proliferation, glomerulomegaly, and proteinuria in control as well as in sickle cell mice (Weber et al., 2008, 2012). All these *in vitro* and *in vivo* studies suggested that morphine has potential to cause mesangial expansion, a precursor of FSGS. However, none of these models represent classical phenotype of HAN.

### Human immunodeficiency virus-associated nephropathy (HIVAN)

A decade after reporting HAN, Rao et al. described another group of patients, exclusively young AAs, both heroin addicts and non-addicts with the Acquired Immunodeficiency syndrome (AIDS), who presented with massive proteinuria, FSGS, rapidly progressing to ESKD (Rao et al., 1984, 1987; Rao and Friedman, 1989). Initially, this entity was referred to as AIDS associated nephropathy, but with the identification of causative agent Human Immunodeficiency Virus (HIV), these authors coined the term HIVAN (Rao and Friedman, 1989). Interestingly, an increase in the incidence of HIVAN was associated with a dramatic decline in new cases of HAN, despite growing number in heroin addiction population (Friedman and Rao, 1995; Monahan et al., 2001). Collapsing form of FSGS is the renal lesion typically seen in patients with HIVAN (D'Agati et al., 1989; Valeri et al., 1996). Numerous studies which analyzed the risk factors for development of renal disease, have shown that more than 90% of patients with HIV associated FSGS are AAs (Bourgoignie et al., 1989; Halevy et al., 2001). Idiopathic FSGS and its collapsing variants are also commonest causes of non-diabetic renal syndromes in AAs (Valeri et al., 1996).

All these observations has led to the speculation that genetic component is a major risk factor for development and progression of renal disease in AAs. However, this concept was a speculation until recent studies which focused on genetic variants are providing valuable evidence in support of this hypothesis.

Even in the experimental models of HIVAN in transgenic mice, susceptibility to kidney disease is influenced by HIV genes and host genetic variants (Rappaport et al., 1994). The initial Tg26 mouse line (model of HIVAN) was generated on the FVB/N inbred mouse strain. This strain was susceptible to develop renal lesions, however, Tg26 mice bred onto the mixed FVB/CAST background mouse strain and others did not develop renal lesions (Gharavi et al., 2004). Genome-wide linkage analysis revealed mouse chromosome 3A1 to 3 region as an HIVAN-susceptibility locus (Chan et al., 2009). Replacement of the FVB/N allele in this locus by the CAST allele induced accelerated disease progression. In addition to Tg26, several other transgenic models have been generated to determine the role of specific HIV genes in the pathogenesis of HIVAN. Expression of an HIV Δgag/pol/nef transgene in mice induced FSGS and microcystic tubular dilation but not podocyte proliferation (Kajiyama et al., 2000); however, when this transgenic mice were back-crossed to a Nef-only transgenic mice, the severity of renal lesions increased (Dickie et al., 1993); these findings indicated the role of Nef and another gene in HIVAN pathogenesis. In another study, in Tg26 mouse model, vpr, nef, or both were additionally deleted; only mice carrying an intact Vpr gene developed FSGS with tubular dilation (Dickie et al., 2004). All these studies indicate the role of specific HIV genes in the pathogenesis in HIVAN.

### Apolipprotein (APO) L1

This gene is located at chromosome 22q13.1, and encodes the minor APOL1 component of High-Density Lipoprotein (HDL). It is expressed in liver, pancreas, kidney, brain, macrophages, endothelial, and several other cell types (Bergman et al., 1996; Duchateau et al., 1997). The G1 variant (rs73885319) is a compound missense mutation (S342G:I384M), encoding two non-synonymous amino acids. G2 variant is a 6 bp in-frame deletion which has resulted in the loss of two amino acids (N388 and Y389) at the C-terminal helix of APOL1 (Dunham et al., 1999; Genovese et al., 2013). Approximately 34% of AAs possess one of the two risk variants and approximately 13% have both coding variants (Dunham et al., 1999; Wasser et al., 2012; Genovese et al., 2013). In the United States, approximately 3 million AAs carry both risk alleles. In contrast, APOL1 risk variants occur infrequently in EAs, approximately 0.3% carry G1 and 0.1% G2 alleles.

### APOL1 and kidney disease

AAs develop higher rates of progressive nephropathy, including idiopathic FSGS, HIVAN, and hypertension-attributed ESKD as compared to European Americans (EAs) (Kopp et al., 2011; Quaggin and George, 2011; Wasser et al., 2012). This disparity is most pronounced in patients with HIV-infection who are not taking highly active antiretroviral drugs. The rate of the development of HIVAN is greater than 10-fold in such untreated subjects (US Renal Data System, 2009). This overwhelming population disparity between AAs and EAs and, greater familial clustering among AA, point to a prominent contribution of underlying genetic factor(s) (Freedman et al., 1999). Recent evidence has shown that this major health disparity is strongly associated with two coding sequence variants (G1 and G2) in APOL1 (Genovese et al., 2010; Tzur et al., 2010; Friedman et al., 2011; Foster et al., 2013). In a recent study, human podocytes expressing APOL1 variants (G1 and G2) displayed enhanced lysosomal swelling and podocyte necrosis (Lan et al., 2014). Moreover, even an overexpression of APOL1 wild type was associated with podocyte toxicity. Interestingly, renal biopsy specimens in patient with APOL1 variants smooth muscle cells displayed enhanced APOL1 expression (Madhavan et al., 2011). These findings prompted us to look into a possibility that smooth muscle may be serving as an endocrine or paracrine source of APOL1 for delivery to podocytes (Lan et al., 2015). This is a plausible hypothesis but needs to be tested in future studies. Since only 4% out of 13% of AAs expressing APOL1 variants develop kidney disease, it appears that in addition to the genetic markers, there has to be additional insult to make podocytes vulnerable to injury. Persistent viral load in the blood and continued exposure to virus accounts for higher rate of HIVAN in this vulnerable population, further supporting this notion.

Renal biopsy studies in patients with FSGS and HIVAN revealed lower expression of APOL1 in podocytes when compared other cells (Madhavan et al., 2011). In a recent report, no correlation was observed between circulating APOL1 levels with HIV infection status or inflammatory mediators in an HIV positive cohort with kidney disease (Bruggeman et al., 2014). Thus, at present it is not clear whether it is locally expressed APOL1 or a burst of circulating APOL1 that contributes to kidney injury. Since there was no relationship between severity of HIV infection and development of kidney disease (Bruggeman et al., 2014), we propose that APOL1- induced vulnerability to HIV effects are more critical for the development of HIVAN rather than the level of viral load.

### Transition of HAN to HIVAN in APOL1Vs bearing population

Disappearance of HAN and appearance of HIVAN in the same population was a puzzle (Friedman and Rao, 1995; Monahan et al., 2001). Investigators who made this observation hypothesized that availability of contaminant-free heroin may account for the disappearance of HAN (Friedman and Rao, 1995). In some earlier studies, Haskell et al. suggested some relationship between HAN and HLA type (Haskell et al., 1988). Recent observations on the role of APOL1 variants in the development of kidney disease in AAs, it appears that heroin may have acted as a trigger for the induction of renal injury in this population. Nevertheless, when the same population was exposed to a stronger and continuous trigger such as HIV infection, the effect of an inconsistent hit such as heroin might have nullified.

## Conclusions

The predominant renal lesion in heroin users is FSGS; however, a variety of other renal lesions have also been reported. The heterogeneity of renal lesions in heroin users points toward the involvement of multiple factors, including social, economic, and genetic. There is no experimental animal model for heroin nephropathy despite multiple attempts by several investigators; this suggests paucity of an ingredient(s) in the existing models. Recent identification of APOL1 variants contributing to disparity in prevalence of FSGS and HIVAN in AAs vs. Caucasians points toward that missing ingredient in experimental animal models (since rodents do not carry APLO1 gene). This also provides an explanation for a decrease in occurrence of HAN with the surge in HIVAN. However, to validate this hypothesis, use of opiates need to be tested in APOL1 transgenic mice in future studies.

### Conflict of interest statement

The authors declare that the research was conducted in the absence of any commercial or financial relationships that could be construed as a potential conflict of interest.
